# How understanding the diversity of perspectives and systems in governments can increase the impact of scientific research

**DOI:** 10.1038/s44528-026-00009-2

**Published:** 2026-07-22

**Authors:** Liza Hadley, Slimane Ben Miled, Hannah E. Clapham, Bimandra A. Djaafara, Louise Dyson, Sebastian Funk, Julia R. Gog, Hans Heesterbeek, Edward M. Hill, Emily Howerton, Valerie Isham, Amira Kebir, Matt Keeling, Stephen M. Kissler, Justin Lessler, Kathy Leung, Emma McBryde, James M. McCaw, Chris Erwin G. Mercado, Denis Mollison, S. M. Thumbi, Wirichada Pan-ngum, Lorenzo Pellis, Francisco J. Pérez-Reche, Michael J. Plank, Robin N. Thompson, Cécile Tran-Kiem

**Affiliations:** 1 University of Colorado Boulder, Boulder, CO USA; 2 University of Cambridge, Cambridge, UK; 3 BioInformatics, bioMathematics and bioStatistics laboratory (BIMS-LR16IPT09), Pasteur Institute of Tunis, University of Tunis El Manar, Tunis, Tunisia; 4 Saw Swee Hock School of Public Health, National University of Singapore and National University Health System, Singapore, Singapore; 5 Oxford University Clinical Research Unit Indonesia, Faculty of Medicine, University of Indonesia, Jakarta, Indonesia; 6 The Zeeman Institute for Systems Biology & Infectious Disease Epidemiology Research, School of Life Sciences and Mathematics Institute, University of Warwick, Coventry, UK; 7 Centre for Mathematical Modelling of Infectious Diseases, London School of Hygiene & Tropical Medicine, London, UK; 8 Department of Population Health Sciences, Utrecht University, Utrecht, The Netherlands; 9 Civic Health Innovation Labs, Department of Public Health, Policy and Systems, Institute of Population Health, NIHR Health Protection Research Unit in Emerging and Zoonotic Infections and The Pandemic Institute, University of Liverpool, Liverpool, UK; 10 Department of Ecology and Evolutionary Biology, Princeton University, Princeton, NJ USA; 11 Department of Statistical Science, University College London, London, UK; 12 IPEIT, University of Tunis, Montfleury, Tunisia; 13 SBIDER, Maths Institute and School of Life Sciences, University of Warwick, Coventry, UK; 14 Department of Epidemiology, Gillings School of Global Public Health, University of North Carolina at Chapel Hill, Chapel Hill, NC USA; 15 The Carolina Population Center, University of North Carolina at Chapel Hill, Chapel Hill, NC USA; 16 Department of Epidemiology, Johns Hopkins Bloomberg School of Public Health, Baltimore, MD USA; 17 The Hong Kong Jockey Club Global Health Institute Pokfulam, Hong Kong SAR, China; 18 WHO Collaborating Centre for Infectious Disease Epidemiology and Control, School of Public Health, LKS Faculty of Medicine, The University of Hong Kong Pokfulam, Hong Kong SAR, China; 19 Laboratory of Data Discovery for Health (D²4H), Hong Kong Science Park Ma Liu Shui, Hong Kong SAR, China; 20 The University of Hong Kong—Shenzhen Hospital, Shenzhen, China; 21 The Hong Kong University of Science and Technology, Hong Kong SAR, China; 22 UQ Centre for Clinical Research (UQCCR), Faculty of Health, Medicine and Behavioural Sciences, The University of Queensland, Brisbane, QLD Australia; 23 School of Mathematics and Statistics, The University of Melbourne, Melbourne, VIC Australia; 24 Centre for Epidemiology and Biostatistics, Melbourne School of Population and Global Health, The University of Melbourne, Melbourne, VIC Australia; 25 Department of Actuarial Mathematics and Statistics, Heriot-Watt University, Edinburgh, UK; 26 Center for Epidemiological Modelling and Analysis, University of Nairobi, Nairobi, Kenya; 27 Institute of Immunology and Infection Research, University of Edinburgh, Edinburgh, UK; 28 Paul G Allen School for Global Health, Washington State University, Pullman, WA USA; 29 Mahidol-Oxford Tropical Medicine Research Unit (MORU) and Department of Tropical Hygiene, Faculty of Tropical Medicine, Mahidol University, Bangkok, Thailand; 30 The University of Manchester, Manchester, UK; 31 School of Natural and Computing Sciences, University of Aberdeen, Aberdeen, UK; 32 Department of Twin Research and Genetic Epidemiology, School of Life Course & Population Sciences, King’s College London, London, UK; 33 School of Mathematics and Statistics, University of Canterbury, Christchurch, New Zealand; 34 Te Pūnaha Matatini, Centre of Research Excellence in Complex Systems, Auckland, New Zealand; 35 Mathematical Institute, University of Oxford, Oxford, UK; 36 Vaccine and Infectious Disease Division, Fred Hutchinson Cancer Center, Seattle, WA USA

**Keywords:** Environmental social sciences, Health humanities, Scientific community, Social sciences

## Abstract

The COVID-19 crisis required scientists worldwide to contribute to complex, hectic, and unfamiliar governmental decision-processes. In this Perspective, we reflect on this intense interaction between science and health policy for pandemic response, drawing from the experience of infectious disease modellers across the world. We highlight the diversity of actors and interests in government, aiming to demystify the elusive ‘policy makers’. We present a general taxonomy to help research scientists more effectively support evidence-based policy. We stress the importance of building and maintaining relationships appropriate to the diverse pool of government actors. Not recognising this diversity may lead to miscommunication and reduce the positive impacts of scientific evidence for public policy in crisis and non-crisis situations.

## Introduction

During the COVID-19 pandemic, the interface between science and government was highly visible and intensely stressed. Scientists worldwide were asked to contribute to a government decision-making process that was hectic, complex, and, for many, unfamiliar. The authors of this Perspective are research scientists who were involved in the pandemic response in 14 different countries across every region of the globe. Through these experiences and other crisis and non-crisis work before and since, we gained important insights into the complex structure of government decision-making and the diversity of actors involved. Building on these experiences and a qualitative study of modelling-to-policy interactions^1^, we present a general taxonomy to help scientists better understand this diversity of actors as they work to effectively support evidence-based policy, both in normal times and times of crisis. This Perspective is aimed primarily at research scientists, especially those who are newly engaging with policy processes or who may do so in future crises. Research scientists are referred to simply as ‘scientists’ in the remainder of our article for brevity.

According to Scopus, from January 1990 to March 2026, over 192,000 scientific articles referenced ‘policy makers’ or ‘decision makers’ in their title, abstract, or keywords. Some may assume that these terms refer to individuals with the ability to make decisions about government or institutional policy, informed in part by scientific information, but more than that is usually left unsaid. The vague abstraction of who in government can use scientific information, and in what ways, hides the true complexity and heterogeneity of real governmental systems and the roles within them. Such simplifications limit scientists’ ability to support evidence-based policy. While such distinctions are well-understood and researched within the scholarly literature on government and public policy (for example, refs. ^2–6^), the day-to-day understanding of scientists of these complexities may limit the impact of their work. Here we argue that it is time for scientists to move beyond viewing ‘government’ as a single, uniform, and abstract entity and instead recognise the diverse interests and roles within it. By doing so, scientists will be able to contribute more effectively to government policy, both during a crisis and in the calmer times between.

*A note on terminology:In this Perspective, we consider a ‘scientist’ to be any individual who identifies primarily or wholly as a scientist, such as those who lead or perform scientific investigations. This article primarily focuses on research scientists/ researchers who use the scientific method to produce new knowledge, findings, or understanding in their respective fields. For this article, we also define ‘policy’ to be a law, regulation, procedure, administrative action, incentive, or voluntary practice of governments and other institutions. Note that ‘science’ and ‘government’ are not always exclusive categories and are not intended as such*.

## The elusive ‘policy maker’

### Who is the ‘policy maker’?

Governments are multi-faceted, complex systems comprising heterogeneous actors each with different objectives, constraints, and timelines. Though it is impossible to capture this complexity with a simple taxonomy, we find it useful to group actors into three categories based on their relationship to the strategy (i.e., broad goals and overall architecture) and tactics (i.e., specific actions and implementation) of government policies:

- Policy specialists, who have working-level (i.e., tactical) responsibility for the specific topic area;
- Policy generalists, who are focused on broader, strategic activities and may not have specialist knowledge in the specific area;
- Decision makers, who are senior public servants and politicians who set overall strategic policy goals and have statutory decision-making powers.

For example, the US CDC’s subject matter experts or Kenya’s public health officers would be ‘policy specialists’; a Chief Epidemiologist for a US state or a Kenyan department Director General would be a ‘policy generalist’; and elected politicians, appointed ministers, or officials with designated statutory authorities and powers would be ‘decision makers’. The distinctions between these groups are not always clear; individuals move between these roles, and there is considerable variation within groups. Individuals may hold different roles, and may move between policy and decision-making roles, or even hold roles with overlapping responsibilities (e.g., when a legislation or regulation delegates a certain decision-making capability to a public servant). Nonetheless, at any given time or in any given context, an individual can be considered to hold a particular role as we have defined it. We argue that ‘policy specialist’, ‘policy generalist’, and ‘decision maker’ are useful categorisations for scientists to consider because the objectives, experience, and remit of each of these actors differ. To facilitate effective dialogue, the nature of the relationships between scientists and each of these actors should therefore also differ.

**Fig. 1:**
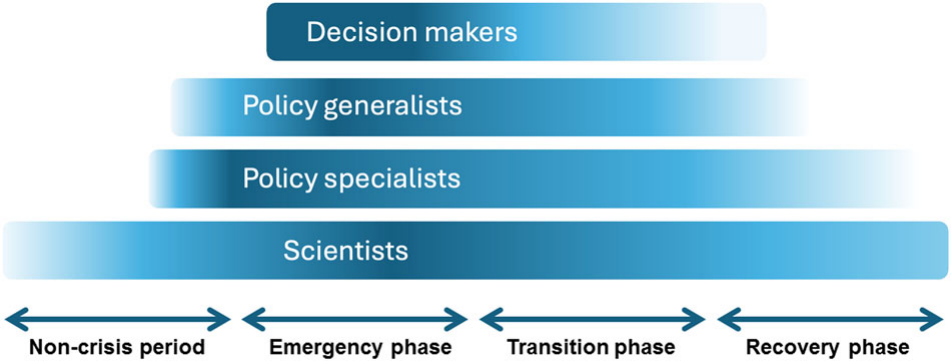
Different actors in government will have a different level of involvement in a health crisis or other emergency depending on the timeline of the threat (horizontal axis represents time). Scientists are typically working on their respective topics at all times, including non-crisis periods. Policy specialists and generalists may be involved on a broad topic in non-crisis periods (e.g. on general preparedness and planning for future emergencies), but may become increasingly engaged with a specific issue as it poses concern or requires monitoring. High-level decision makers are likely to be directly engaged only when the issue poses an acute high-level threat or emergency, with little to no involvement with scientists and researchers in recovery and non-crisis periods. Interaction pathways between actors are not shown - readers interested in this are directed to, for example, refs. ^1,7,44,45,49–51^, which investigate these complex processes in the context of COVID-19.

For any particular science-policy issue, the level of government actors involved will likely depend on the event’s acute impact and where it sits in the scale of political importance (Fig. 1). Much of the time, detailed exchanges may only be between scientists and policy specialists. When the threat level is higher, scientists may be requested to engage directly with policy generalists and decision makers. These interactions can occur either organically or through the explicit creation or reassignment of groups that include government actors with a mix of roles in response to the crisis. Two examples are illustrative: (1) In mainland China before the COVID-19 pandemic, academic scientists worked with infectious disease policy specialists at the Chinese Centre for Disease Control and Prevention (China CDC). However, a separate deputy-ministerial-level agency called the National Administration of Disease Control and Prevention was convened in 2021 in response to the COVID-19 pandemic. In this agency, academic scientists had direct contact with policy generalists. (2) Similarly, in Tunisia, government institutions like the National Observatory of New and Emerging Diseases focus on long-term surveillance and preparedness during non-crisis periods, facilitating interaction between academic scientists and policy specialists. During the COVID-19 pandemic, distinct rapid-response bodies like the National Coronavirus Response Authorities were activated to coordinate efforts across regions and sectors; these entailed more direct interaction between academic scientists and policy generalists. Post pandemic, responsibility for communicable disease management has largely returned to specialist expert bodies, in China, Tunisia, and many other countries.

There may be potential risks and challenges to consider when policy specialists are not directly involved, as in the examples above. For instance, policy generalists and decision makers may be inexperienced in understanding the language and scientific principles of a field, leading to the misinterpretation of evidence and recommendations. Likewise, the experience and knowledge gained by high-level generalists over the course of a crisis may be lost once they return to other priorities. Lessons may not become embedded in post-crisis business-as-usual practices.

Although the above and subsequent examples in our article are focused on national-level government, the types of actors considered here are present in government at all geographic scales - though scientists should be attentive to which of these scales of decision making carries the force of law. What differs most is the size of actor groups and the populations and local issues they are faced with. Structural heterogeneity in science-policy between countries is examined in detail in, for example, Hadley et al. ^1^. However, across different countries and levels of government, the need to tailor research outputs and communicate differently with specific actors such as specialists, generalists, and decision makers remains.

### Building and maintaining science-policy relationships

Communications between scientists and government actors need to be tailored to the attention, level of detail, and turnaround time required by different government actors. Relationships with policy specialists will often create the personal ‘who am I going to call’ networks and crucial trust in information sharing. Sustained relationships with policy specialists not only deliver benefits during crises, but support non-crisis activities, through development of in-house scientific capacity, more targeted foundational research, and more rapid adoption of scientific advances into policy^1,7–12^. In contrast, relationships with policy generalists and decision makers typically centre on broad strategic relevance and also enable familiarity with particular types of scientific outputs. These actors may engage with specific scientific subfields less frequently or with less technical depth, so scientists must adjust their language accordingly. While some policy specialists may be comfortable with technical language, there are different language needs for decision makers. At times, certain terms can carry specific regulatory meanings (such as ‘activate’ in a US regulatory context, or ‘probable’ within UK government probability frameworks^13^). Points that seem elementary to those working in the field may not be well known to policy generalists and decision makers with different expertise, and should be explicitly stated. Preferences around format and timescale for analyses also differ^14–16^. ‘Stepped’ reports—presenting a high-level summary for decision makers, a short report for generalists, and a detailed analysis for specialists—may help bridge these gaps. The communication of uncertainty is a particular challenge, as the languages of scientists and policy actors frequently diverge in this area^17^. How best to overcome the challenge of communicating uncertainty has been the source of much research and debate, and scientists should familiarise themselves with this background^18^. We provide examples to illustrate how relationships and routes of communication may differ when working with policy specialists, policy generalists, or decision makers (Case studies box and Table 1).

**Table 1:** Who do scientists interact with in government?

	Example roles	Case studies from communicable disease epidemiology
**Policy specialists**	Health officer, public health agency subject expert.	In Indonesia, long-term collaborations between academic epidemiological modellers and policy specialists in Dengue and Malaria Control enabled direct requests to come through to scientists, with direct interactions with each disease sub-directorate in the Ministry of Health. Engagements included discussions, co-creation of questions, and two-way feedback through regular calls and physical meetings. Similar routes of communication are seen in Thailand, where modellers could engage with specialist policy during the pandemic through existing (non-crisis) collaboration within the Emerging and Re-Emerging Infection Disease programme, part of the Ministry of Science and Technology.
**Policy generalists**	General public sector policy maker, department Director Generals, chief scientific advisors, chief medical officers.	In Kenya, scientists at the Centre for Epidemiological Modelling and Analysis (CEMA) together with modelling teams in other in-country academic and research institutions, worked most closely with the Director General for Health (a policy generalist), helping the Minister of Health with planning of interventions based on locally-generated data. In China during the COVID-19 pandemic, academic modellers supported senior actors in the China CDC (policy generalists), where they were asked for ad hoc reporting and to communicate directly with the CDC when discussions were needed. Most reports were concise (<1000 characters) or in powerpoint format. During the Omicron wave, these modellers were also requested to visit the CDC to provide real-time reporting. In New Zealand, this short line of communication to decision makers similarly needed a brief, concise summary of results (see case studies box). As a more nuanced example, the World Health Organisation regional offices for South-East Asia (SEARO) and the Western Pacific (WPRO) facilitated links with policy generalists in countries that had limited scientific capacity, connecting academic modellers from Australia directly to Chief Medical Officers in Malaysia, Indonesia, Fiji, and French Polynesia.
**Decision makers**	Prime minister or president, governors, ministers.	A few academic modellers in Australia and the UK were seconded to their national governments’ Cabinet (the senior decision-making level) or sub-committee during COVID-19, where they played a primarily interpretative role. There were often faster turnaround times than working with policy specialists, and most of the work in this example involved interpreting and translating a broad literature. Here, it was clear that communicable disease epidemiology formed one part of a much larger picture. In South Africa, modellers played a similar role through appointment to the Ministerial Advisory Committee on COVID-19, where they worked in an interdisciplinary group to provide policy advice to the Minister of Health in response to questions on a wide variety of topics. In Singapore, COVID-19 results for decision makers were primarily distilled into key PowerPoint slides providing the ‘big picture’ findings (in contrast to working with policy specialists, where the academic modellers could discuss methods, model structure, parametrisation, and future data needs directly).

Examples of how relationships may differ when interacting with different government actors, such as policy specialists vs policy generalists vs decision makers.

Going deeper, relationships between science and government require both tangible ‘hardware’ such as data sharing agreements, memorandums of understanding, and advisory group membership, as well as intangible ‘software’ such as mutual values, norms, relationships, and trust^19^. The hardware underpinning these relationships is particularly important to policy specialists, who need legal support for sharing information and often work within strict regulatory frameworks. It is critical to maintain both hardware and software during non-crisis periods to enable rapid scale-up when new threats emerge. For example, several COVID-19 modelling groups supported by government funding agencies in Australia benefited from existing trusted relationships with policy generalists in the Commonwealth and State Health departments (established software), but, without critical agreements in place, data-sharing was slow and structural capacity to build consensus through open science was insufficient (lacking hardware). The crisis catalysed change, including commitment to establish an Australian Centre for Disease Control (a home for policy specialists with established scientific relationships)^20^, but many of these changes only materialised post-crisis. Scientists must approach the establishment of such relationships or agreements with the government with an awareness that decision makers and other elected government officials may view the response to topics in the public eye through a political lens, while for scientists, the independence from such matters is paramount. Scientific independence helps ensure research findings are robust, with implications for public trust and the long-term sustainability of science-policy relationships.

Another important consideration is personnel turnover and shifting attention in government, which makes one-to-one relationships fragile. As highlighted in Fig. 1, different levels of actors may be focused on a particular topic at different times. In STEMM disciplines (Science, Technology, Engineering, Mathematics and Medicine), attrition is high, particularly among early-career researchers (e.g. approximately 58% cease publishing within nine years)^21^. Equally, for the UK government, general policy, for example, several departments experience more than 10% of civil servants either moving between departments or leaving the civil service each year^22,23^. These statistics do not include moves within government departments or between topics of interest, meaning overall civil service ‘churn’ is likely even higher. High rates of turnover among public servants are also reported anecdotally in other countries, including both HICs and LMICs. This erosion of institutional memory (on either side) poses a significant challenge for preparedness: the informal knowledge, working relationships, and mutual understanding built between scientists and government actors during a crisis are not always formally documented and can be difficult to reconstruct when the next threat emerges^24–27^. This motivates an approach to engagement which is constantly establishing and renewing relationships between individuals, institutions, agencies, and groups. Examples of facilitating such activities through hardware include: systematic documentation of interactions and relationship-building processes; academic representation on committees; active involvement in general/specialist policy forums; dual appointments/secondments; and co-funded research by governments, where possible, to create a sense of ownership and independence from external/foreign funding where relevant.

Case studies box: **“How do relationships differ with policy specialists vs policy generalists vs decision makers?”**

**#1 Feedback**. Often more detailed feedback is available on research when interacting with policy specialists compared to decision makers. In France during the COVID-19 pandemic, some modellers had well-established procedures with the National Immunisation Technical Advisory Group and the Haute Autorité de Santé (HAS), the French agency responsible, among other things, for vaccine policy recommendations. The presence of a project manager within HAS enabled frequent back-and-forth and feedback from policy specialists, involving regular interactions and co-creation of research questions. In the United States, the US Scenario Modelling Hub worked with a range of policy actors during the pandemic, including policy generalists (e.g. White House Coronavirus Task Force), policy specialists (e.g. the Advisory Committee on Immunisation Practices (ACIP), part of the US CDC), as well as state level public health officials primarily through interactions with individual teams. The strongest feedback loop again emerged with policy specialists, where model scenarios were designed specifically around questions posed by specialists at the US CDC, and eventually these projections were cited in ACIP decisions.

**#2 Level of detail**. In New Zealand, early on in COVID-19, modellers interacted mainly with Chief Scientific Advisors (policy generalists) who communicated their results directly to decision makers. This direct line of communication to decision makers needed a short, concise summary of results, e.g. a simple graph or soundbite, or a one-sentence response to a question over the phone. Sometimes graphs or other material were also requested for sharing at press conferences. As the focus moved to more specialist policy (health policy advisors), modellers were more likely to be asked for detailed sensitivity analysis, comparison of multiple scenarios and policy combinations, etc. These details are of course still important when interacting with senior decision makers, but the latter are more likely to want to see a high-level, very non-technical summary than a detailed set of results.

**#3 Connecting different questions**. In the UK, policy specialists were able to connect a current government research request on whether to change self-isolation strategies so that people could leave isolation early if they received a negative Lateral Flow Test each morning, with previous research completed months earlier on different testing strategies for reopening schools. This enabled existing models to be used to help answer the new question. In this scenario, policy specialists working closely with the scientists were able to make connections that other actors within government (i.e. policy generalists and decision makers) who were less familiar with the underlying methods could not.

**#4 Structured pathways to decision makers**. Direct interactions between scientists and high-level decision makers can occur, and the structure of such interactions varies greatly. One common structure is to appoint a scientist to a national-level liaison role, such as the chief scientific advisor and the chief medical officer in the UK. The Netherlands previously had formal structures for infectious disease modelling evidence to reach the Prime Minister via the head of the Outbreak Management Team during outbreaks. Note, however, that in the case of COVID-19 in the Netherlands, this advice came solely from modellers within the national government institute for health (RIVM), owing to the absence of wider institutional mechanisms that would allow interaction and input from the broader academic scientific community. These are limitations that RIVM has since begun to address in its post-pandemic planning.

**#5 Small populations**. Modelling support in French Guyana and Wallis and Futuna, two French overseas territories, saw a special case of science-government interaction where meetings gathered academic modellers, policy specialists, and senior decision makers all together. Similarly, in the Isle of Man, a British overseas territory with a similarly small population and governmental structure, one team of modellers conversed directly with the Council of Ministers (decision makers) to aid their decisions on isolation strategy.

*Box: Case studies illustrating how the relationships between scientists and government actors need to be nuanced to the attention, level of detail, and turnaround required by different policy levels*.

## What else can scientists do?

Appropriate and nuanced relationships between scientists and different government actors are the most important ingredients if science is to contribute to effective preparedness and response. Underpinning these relationships is sustained government investment in scientific capacity, including research funding, embedded scientific roles, and career pathways that enable scientists to engage with policy over the long term. For example, the UK and Australia developed formal epidemiological modelling capability in the early 2000s with continual engagement to date, meaning that science-policy systems, while not perfect, were already established when new threats emerged. In contrast, Hong Kong’s epidemiological modelling capacity grew gradually after the 2003 SARS epidemic but was not routinely integrated into seasonal epidemic or pandemic response until COVID-19, complicating the science-policy interface. Other countries had long-established disease-specific linkages, such as the use of the Thembisa model for HIV/AIDS planning in South Africa^28^, while having little experience of incorporating modelling into decision-making for outbreaks of respiratory diseases.

Training (for both research scientists and government actors) is important for getting the most out of these relationships due to the differences in primary responsibilities and focus^29^. While much of this learning happens informally, we suggest three specific actions for external scientists seeking to actively engage in policy:

1. Firstly, it is crucial for scientists to learn (and adopt) the *language* and communication culture of the different government actors we are working with, as discussed in Section 2.2^13–15,17,18,30^. Concrete pathways for developing this familiarity exist in many settings, including policy fellowships, short placements in government organisations, and initiatives run by learned societies and science-policy intermediaries. Examples of broad training courses for scientists include the American Association for the Advancement of Science (AAAS) federal policy fellowships in the US, the Wellcome-led “Science Policy: Improving the Uptake of Research into UK Policy” residential course in the UK, and more recently, the ASEAN Science Diplomacy workshop held in Thailand^31–33^. As a more specific public health example, the South African Centre for Epidemiological Modelling & Analysis (SACEMA) supports Policy Modelling Fellowships^34^. Similarly, the Global Health Network (a World Health Organisation “Collaborating Centre” for research information sharing, e-learning, and capacity development) acts as a free, online knowledge hub to share research findings, bolster the uptake of research, and foster a community of practice for researchers, government actors, and development partners^35^. Science Media Centres and comparable organisations in several countries also play an invaluable role in providing advice and training for scientists engaging with media and connecting researchers with journalists^36^. There are many more excellent examples, and we advocate for each scientific field to clearly signpost the opportunities for science-policy training and language development in their field-specific journals and conferences.
2. In the same way that governments can be difficult for scientists to navigate, the science community can be difficult for government actors to navigate. In countries fortunate enough to have multiple experts engaged on a given topic, we argue that a diversity of perspectives should be actively solicited, discussed, challenged, and refined by the relevant scientific groups^37,38^. Learned societies can provide one option for aiding solicitation and enabling a forum for new scientists who want to contribute^11^. However, as far as possible, views should be communicated to decision makers, and to a lesser extent, policy generalists, as a coherent body of evidence. In some contexts, it may be preferable for this distillation and translation of evidence to be performed within government by policy specialists, by scientists and policy specialists collaboratively, or by independent intermediaries^1,39,40^. The latter was seen in some COVID-19 settings in Thailand, where contacts with epidemiological modellers were mainly indirect through experts and senior academic staff advising policy generalists and decision makers.
3. Lastly, ‘decision science’ could provide a pathway for translating scientific endeavour to evidential support for actions. Doing so would require a subset of researchers to follow this methodology and support government actors (particularly policy generalists) in framing their problems in ways that these techniques can be applied. Suggestions of how to apply a structured decision science approach have been recently outlined in a few fields, for example, in infectious diseases, conservation, and climate science^41–43^. By acknowledging that the decision process must also account for social, political, and organisational factors, the contribution of science to decision-making can be better understood.

## Concluding remarks

We have sought to illustrate how relationships between science and government may differ depending on the type of government actor involved (Fig. 1, Table 1, Case studies box). Previous work has provided valuable accounts of science-policy structures in specific national settings (for example^44–48^), whereas our contribution draws on experience across 14 countries with diverse governance contexts. In addition to different types of government actors (Fig. 1), there are also significant differences between systems and levels of government, for example, national versus local government, and federal versus unitary systems. Heterogeneity within and between jurisdictions in factors such as political systems, institutional arrangements, and existing science–government linkages also affects how science advice can operate in practice. We do not aim to advance a one-size-fits-all solution to scientific advice, but rather identify common themes and needs of different government actors. The extent to which these themes and needs are applicable or whether other considerations dominate will differ according to context.

Nonetheless, if scientists do not acknowledge the diversity of actors in government decision-making and oversimplify their understanding of the decision-making processes, it may lead to miscommunication and a reduced impact of scientific evidence in public policy. The elusive ‘policy maker’ will remain so. Conversely, a greater understanding and explicit consideration of the diversity of actors within government can support more impactful contributions of science to policy in emergency and routine decision-making.

## Data Availability

No datasets were generated or analysed during the current study.

## References

[1] L. Hadley, C. Rich, A. Tasker, O. Restif, S. Funk, How does policy modelling work in practice? A global analysis on the use of modelling in Covid-19 decision-making, PLOS Glob. Public Health. 10.1371/journal.pgph.0004675 (2025). 10.1371/journal.pgph.0004675 PMC12143527 40478854

[2] Knill, C. Explaining cross-national variance in administrative reform: autonomous versus instrumental bureaucracies. *J. Public Policy* **19**, 113–139 (1999).

[3] F. M. Van Der Meer, J. C. N. Raadschelders, T. A. J. Toonen, eds., Comparative civil service systems in the 21st century. 10.1057/9781137491459 (Palgrave Macmillan, 2015).

[4] F. R. Baumgartner, B. D. Jones, Agendas and Instability in American Politics. (University of Chicago Press, 2010).

[5] F. F. Ridley, ed., Specialists and generalists: a comparative study of the professional civil servant at home and abroad. 10.4324/9781003476610 (Routledge, 2024).

[6] Cairney, P. & Kwiatkowski, R. How to communicate effectively with policymakers: combine insights from psychology and policy studies. *Palgrave Commun.* **3**, 37 (2017).

[7] Oliwa, J. et al. Framework to guide the use of mathematical modelling in evidence-based policy decision-making. *BMJ Open* **15**, e093645 (2025). 40187784 10.1136/bmjopen-2024-093645 PMC11973756

[8] Dilling, L. & Lemos, M. C. Creating usable science: opportunities and constraints for climate knowledge use and their implications for science policy. *Glob. Environ. Change* **21**, 680–689 (2011).

[9] C. Craig, How does government listen to scientists? / C. Craig., Palgrave Macmillan, Cham, Switzerland. https://cam-ldls.lib.cam.ac.uk/ark:/81055/vdc_100073362888.0x000001 (accessed March 26, 2025), 2019.

[10] P. C. Martin, D. H. Kan, M. Fink, Crisis preparation in the age of long emergencies, Blavatnik School of Government, (University of Oxford, 2023).

[11] Dangerfield, C. E. et al. Getting the most out of maths: How to coordinate mathematical modelling research to support a pandemic, lessons learnt from three initiatives that were part of the COVID-19 response in the UK. *J. Theor. Biol.* **557**, 111332 (2023). 36323393 10.1016/j.jtbi.2022.111332 PMC9618296

[12] Ettinger, J. et al. Communicating with policy makers about climate change, health, and their intersection: a scoping review, Lancet Planet. *Health* **9**, e53–e61 (2025). 10.1016/S2542-5196(24)00307-3 39855234

[13] Professional Head of Intelligence Assessment, The PHIA Probability Yardstick. Appendix to: Professional Development Framework for all-source intelligence assessment. https://www.gov.uk/government/publications/intelligence-analysis-professional-development-framework (PHIA, 2019).

[14] Hadley, L., Rich, C., Tasker, A., Restif, O., Funk, S. Visual preferences for communicating in crises: evidence from public health decision makers 2024.11.05.24316774 10.1101/2024.11.05.24316774 (2024).

[15] CMCC Policy Group, Guidance on use of modelling for policy responses to COVID-19. https://www.hitap.net/en/document/covid19-policy-modelling-guidance/ (2020).

[16] J. McVernon, et al. Improving the communication of scientific results to policy makers, Zenodo. 10.5281/zenodo.15331321 (2014).

[17] Kinzig, A. & Starrett, D. Coping with uncertainty: a call for a new science-policy forum. *AMBIO J. Hum. Environ* **32**, 330–335 (2003). 10.1579/0044-7447-32.5.330 14571961

[18] van der Bles, A. M. et al. Communicating uncertainty about facts, numbers and science, R. Soc. *Open Sci* **6**, 181870 (2019). 10.1098/rsos.181870 PMC6549952 31218028

[19] Palagyi, A. et al. Health system preparedness for emerging infectious diseases: a synthesis of the literature, Glob. *Public Health* **14**, 1847–1868 (2019). 10.1080/17441692.2019.1614645 31084412

[20] Australian Government Department of Health and Aged Care, Australian Centre for Disease Control. https://www.health.gov.au/our-work/Australian-CDC (accessed April 4, 2025), (2023).

[21] M. Kwiek, L. Szymula, Quantifying attrition in science: a cohort-based, longitudinal study of scientists in 38 OECD countries, High. Educ. 10.1007/s10734-024-01284-0. (2024).

[22] Cabinet Office, Civil Service statistics, GOV.UK. https://www.gov.uk/government/collections/civil-service-statistics (accessed November 22, 2024) (2024).

[23] T. Sasse, E. Norris, Moving On: The costs of high staff turnover in the civil service. https://www.instituteforgovernment.org.uk/sites/default/files/publications/IfG_staff_turnover_WEB.pdf (2019).

[24] Tsuei, S. H.-T. How previous epidemics enable timelier COVID-19 responses: an empirical study using organisational memory theory. *BMJ Glob. Health* **5**, e003228 (2020). 32967981 10.1136/bmjgh-2020-003228 PMC7513424

[25] Corbett, J., Grube, D. C., Lovell, H. & Scott, R. Singular memory or institutional memories? Toward a dynamic approach. *Governance* **31**, 555–573 (2018).

[26] Stark, A. Explaining institutional amnesia in government. *Governance* **32**, 143–158 (2019).

[27] K. Smith, Institutional Amnesia and the Rise of Public Health Knowledge Brokers, in: K. Smith (Ed.), Evid.-Based Policy Public Health Interplay Ideas, pp. 201–212. 10.1057/9781137026583_7 (Palgrave Macmillan, 2013).

[28] L. Johnson, et al. Thembisa Project. https://www.thembisa.org/ (accessed March 17, 2026), (Thembisa, 2022).

[29] Nature Advising governments about science is essential but difficult. So train people to do it. *Nature* **636**, 8–8 (2024). 39633200 10.1038/d41586-024-03910-4

[30] M. McMahon, M. Naylor, Getting through: communicating complex information. 10.2139/ssrn.4698260 (SSRN, 2024).

[31] AAAS, Science & Technology Policy Fellowships. https://www.aaas.org/programs/science-technology-policy-fellowships (accessed December 2, 2024), (AAAS, 2023).

[32] Wellcome Connecting Science Courses and Conferences, Science Policy: Improving the Uptake of Research into UK Policy. https://coursesandconferences.wellcomeconnectingscience.org/event/science-policy-improving-the-uptake-of-research-into-uk-policy-20230821/ (accessed February 4, 2025), (2025).

[33] Thailand Science, Research and Innovation, Science Diplomacy Workshop for Early-to-Mid Career Researchers in ASEAN Plus Three, n.d. https://www.belmontforum.org/wp-content/uploads/2024/07/%EB%B6%99%EC%9E%841._Call-pilot_Science_Diplomacy_Workshop.pdf (accessed February 4, 2025).

[34] SACEMA, Policy Modelling Fellowships. https://www.sacema.org/opportunities/policy-modelling-fellowships/ (accessed February 4, 2025), (SACEMA, 2025).

[35] The Global Health Network, Home • The Global Health Network. https://tghn.org/ (accessed November 22, 2024), (2024).

[36] Rödder, S. Science media centres and public policy. *Sci. Public Policy* **42**, 387–400 (2015).

[37] Shea, K. et al. Harnessing multiple models for outbreak management. *Science* **368**, 577–579 (2020). 32381703 10.1126/science.abb9934

[38] A. Boin, P. ’t Hart, E. Stern, B. Sundelius, The politics of crisis management: public leadership under pressure, Cambridge University Press, Cambridge. 10.1017/CBO9780511490880 (2005).

[39] Owek, C. J. et al. Lessons learned from COVID-19 modelling efforts for policy decision-making in lower- and middle-income countries. *BMJ Glob. Health* **9**, e015247 (2024). 39521455 10.1136/bmjgh-2024-015247 PMC11552008

[40] P. Christen, S. Van Elsland, D. Saulo, A. Cori, J. Fitzner, Workshop report: advanced analytics to inform decision making during public health emergencies, [object Object], WHO Hub for Pandemic and Epidemic Intelligence, Berlin, Germany. 10.25561/108600 (2023).

[41] V. Hemming et al. An introduction to decision science for conservation. 10.1111/cobi.13868 (2021). 10.1111/cobi.13868 PMC9302662 34856010

[42] C. M. Baker et al. From climate change to pandemics: decision science can help scientists have impact. *Front. Ecol. Evol.* 10. 10.3389/fevo.2022.792749 (2022).

[43] F. M. Shearer, R. Moss, J. McVernon, J. V. Ross, J. M. McCaw, Infectious disease pandemic planning and response: Incorporating decision analysis. *PLOS Med.* **17**, e1003018 (2020). 10.1371/journal.pmed.1003018 PMC6952100 31917786

[44] van Elsland, S. L. et al. Policy impact of the Imperial College COVID-19 Response Team: global perspective and United Kingdom case study. *Health Res. Policy Syst* **22**, 153 (2024). 39538321 10.1186/s12961-024-01236-1 PMC11559147

[45] van Kleef, E. et al. Modelling practices, data provisioning, sharing and dissemination needs for pandemic decision-making: a European survey-based modellers’ perspective, 2020 to 2022. *Eurosurveillance* **30**, 2500216 (2025). 41133306 10.2807/1560-7917.ES.2025.30.42.2500216 PMC12555114

[46] Aguas, R. et al. Modelling the COVID-19 pandemic in context: an international participatory approach. *BMJ Glob. Health* **5**, e003126 (2020). 33361188 10.1136/bmjgh-2020-003126 PMC7759758

[47] Sherratt, K. et al. Improving modelling for epidemic responses: reflections from members of the UK infectious disease modelling community on their experiences during the COVID-19 pandemic. *Wellcome Open Res.* **9**, 12 (2024). 38784437 10.12688/wellcomeopenres.19601.1 PMC11112301

[48] Jit, M. et al. Reflections on epidemiological modeling to inform policy during the COVID-19 pandemic in Western Europe, 2020–23. *Health Aff.* **42**, 1630–1636 (2023). 10.1377/hlthaff.2023.00688 38048502

[49] Le Rutte, E. A. et al. A case for ongoing structural support to maximise infectious disease modelling efficiency for future public health emergencies: a modelling perspective. *Epidemics* **46**, 100734 (2024). 38118273 10.1016/j.epidem.2023.100734

[50] Jit, M. How modelling can better support public health policy making: the Lancet Commission on Strengthening the Use of Epidemiological Modelling of Emerging and Pandemic Infectious Diseases. *Lancet*10.1016/S0140-6736(23)02758-7 (2023). 10.1016/S0140-6736(23)02758-7 38141627

[51] Brooks-Pollock, E., Danon, L., Jombart, T. & Pellis, L. Modelling that shaped the early COVID-19 pandemic response in the UK. *Philos. Trans. R. Soc. B Biol. Sci.* **376**, 20210001 (2021). 10.1098/rstb.2021.0001 PMC8165593 34053252

